# Ribosomal frameshifting used in influenza A virus expression occurs within the sequence UCC_UUU_CGU and is in the +1 direction

**DOI:** 10.1098/rsob.120109

**Published:** 2012-10

**Authors:** A. E. Firth, B. W. Jagger, H. M. Wise, C. C. Nelson, K. Parsawar, N. M. Wills, S. Napthine, J. K. Taubenberger, P. Digard, J. F. Atkins

**Affiliations:** 1 Division of Virology, Department of Pathology, University of Cambridge, Cambridge CB2 1QP, UK; 2 Viral Pathogenesis and Evolution Section, Laboratory of Infectious Diseases, National Institute of Allergy and Infectious Diseases, National Institutes of Health, Bethesda, MD 20892, USA; 3 The Roslin Institute, University of Edinburgh, Easter Bush, Midlothian EH25 9RG, UK; 4 Mass Spectrometry and Proteomics Core Facility, University of Utah, Salt Lake City, UT 84112, USA; 5 Department of Human Genetics, University of Utah, Salt Lake City, UT 84112, USA; 6 BioSciences Institute, University College Cork, Cork, Republic of Ireland

**Keywords:** genetic recoding, ribosomal frameshifting, mass spectrometry, influenza virus, PA-X, translation

## Abstract

Programmed ribosomal frameshifting is used in the expression of many virus genes and some cellular genes. In eukaryotic systems, the most well-characterized mechanism involves –1 tandem tRNA slippage on an X_XXY_YYZ motif. By contrast, the mechanisms involved in programmed +1 (or −2) slippage are more varied and often poorly characterized. Recently, a novel gene, PA-X, was discovered in influenza A virus and found to be expressed via a shift to the +1 reading frame. Here, we identify, by mass spectrometric analysis, both the site (UCC_UUU_CGU) and direction (+1) of the frameshifting that is involved in PA-X expression. Related sites are identified in other virus genes that have previously been proposed to be expressed via +1 frameshifting. As these viruses infect insects (chronic bee paralysis virus), plants (fijiviruses and amalgamaviruses) and vertebrates (influenza A virus), such motifs may form a new class of +1 frameshift-inducing sequences that are active in diverse eukaryotes.

## 2. Introduction

During translation, shifts in reading register can occur to either alternative frame. The most widely known frameshifting mechanism involves shifting to the –1 frame. In part, this is because of the relatively well-defined nature of the most commonly used shift site motif that allows two adjacent tRNAs to re-pair to mRNA in the –1 frame, and in part due to the prominence of the viruses and other mobile elements that use this type of frameshift. The other reading frame can be accessed by either a –2 or a +1 frameshift event, with the product of the former having an extra amino acid encoded by the shift site sequence relative to the latter.

In the majority of bacteria, frameshifting to the +1 frame is used as a sensor and effector of an autoregulatory circuit for the expression of release factor 2 [1,2]. In animals and fungi, such frameshifting is widely used to regulate expression of antizyme, the negative regulator of cellular polyamine levels [3,4]. In both cases, protein sequencing has shown that the shift is +1. Interestingly, however, although the mammalian antizyme 1 frameshifting signals exclusively drive +1 frameshifting in mammalian cells, they induce both +1 and –2 frameshifting when a cassette containing them is expressed in *Schizosaccharomyces pombe*, and –2 frameshifting when expressed in *Saccharomyces cerevisiae* [5]. In this system, the ratio of –2 to +1 is alterable depending on the distance of a 3′-adjacent stimulatory pseudoknot structure from the shift site [6]. Similarly, frameshifting on the HIV shift site U_UUU_UUA, which is normally –1, can be altered to –2 by varying the distance to the 3′ stimulatory element [7]. The only known natural case of programmed –2 frameshifting occurs during the expression of the gpGT tail assembly protein of phage Mu, where the efficiency of frameshifting is estimated to be about 2.2 per cent [8,9]. Protein sequencing has also been used to determine that +1 frameshifting is used in the expression of the *Tsh* gene of several *Listeria* phages and *Bacillus subtilis* SPP1 phage, besides *Escherichia coli yepP*, and the *pol* gene of the *S. cerevisiae* retrotransposons Ty1 and Ty3 [10–14]. Given similar sequences as in Ty1, the frameshifting used in decoding the mRNAs for actin filament binding protein ABP140 and telomere component EST3 is also expected to be +1 [15–18].

Frameshifting, probably in the +1 direction, has also been reported in mitochondria from several diverse species, although functionally different cases of frameshifting used in human mitochondria are –1 [19–22]. Peptide analysis has confirmed shifting to the +1 frame in one of the significant number of *Euplotes* genes that use such frameshifting, but the transframe-encoded peptide that would demonstrate the nature of the shift remains elusive [23,24]. Further work is also required on the early identified case involving the RNA phage MS2 coat lysis hybrid [25]. Low-efficiency cases are especially challenging—for instance, that of the clinically relevant shifting to the +1 frame that is seen in some cases of drug-resistant herpes simplex virus [26–28]. As a test case, even very low levels of the resulting frameshift product were shown to be able to function as an epitope for stimulation of CD8+ T cells [29].

**Figure 1.**
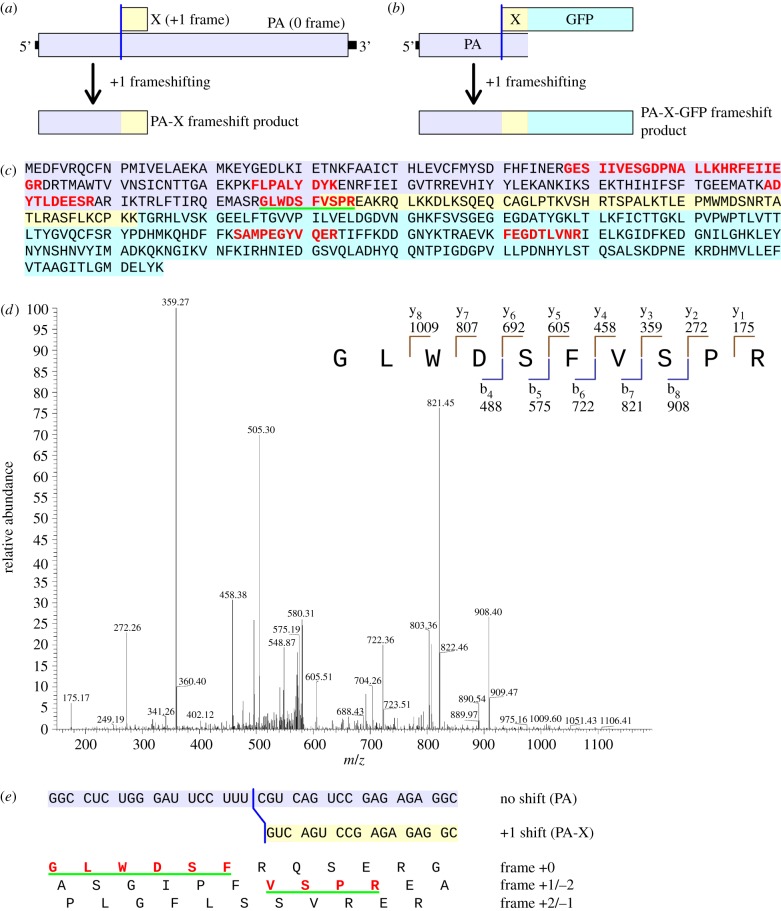
Mass spectrometric analysis of the PA-X-GFP frameshift fusion protein. (*a*) Translation map of influenza A virus segment 3 showing full-length PA and the transframe fusion PA-X that comprises the N-terminal domain of PA fused to a C-terminal tail encoded by the +1 reading frame. (*b*) Map of the construct used to purify the product of frameshifting on the PA-X frameshift cassette. (*c*) Complete amino acid sequence of PA-X-GFP. Amino acids encoded by the zero-frame are highlighted in mauve; amino acids encoded by the +1 frame are highlighted in pale yellow (X) or cyan (GFP). The eight peptides identified by mass spectrometry are indicated in red (note that the sequence GES…EGR corresponds to three detected peptides GES…LLK, HRF…EGR and FEI…EGR). The peptide spanning the frameshift site is underlined in green. (*d*) MS/MS fragmentation spectrum of the shift site peptide GLWDSFVSPR. The inset shows the peptide sequence with ‘b-’ and ‘y’-type fragment ions that strongly support the shift site peptide identified in the nano-LC/MS/MS analysis. Several additional fragment ions, corresponding to H_2_O losses from b and y series ions and doubly charged fragment ions, are also present in the spectrum to further support the sequence (assignments not labelled in the figure). (*e*) Nucleotide sequence in the vicinity of the frameshift site UCC_UUU_CGU, with conceptual amino acid translations in all three reading frames. The product of +1 frameshifting is indicated in red. The green-underlined peptide, which spans the shift site, is compatible with +1, but not –2, frameshifting.

Recently, Jagger *et al*. identified a novel coding ORF (X) in influenza A virus [30]. The X ORF is translated as a transframe fusion (PA-X) with the N-terminal domain of the PA protein (figure 1*a*). PA is a component of the viral polymerase, and the N-terminal domain carries an endonuclease activity that, as part of PA, cleaves capped RNA fragments from cellular pre-mRNAs to act as primers for viral transcription [31]. As PA-X, however, the N-terminal domain appears to play a role in host cell shut-off, presumably by cleaving host mRNAs. PA-X expression depends on ribosomal frameshifting into the +1 frame, and comparative sequence analysis suggests that the frameshifting occurs within a highly conserved UCC_UUU_CGU sequence at the 5′ end of the X ORF (underscores separate zero-frame, i.e. PA, codons) [30]. However, the exact site and direction of frameshifting was not determined. Here, we identify the nature of the shift to the +1 frame in PA-X expression. The results highlight the coding versatility of the sequence UCC_UUU_CGU, with expression relevance for genomes (both viral and cellular) less well studied than influenza A virus.

## 3. Results and discussion

The efficiency of frameshifting at the PA-X shift site was previously estimated by translating reporter constructs in rabbit reticulocyte lysates and found to be around 1.3 per cent [30]. When the frameshift cassette was fused into a dual luciferase reporter construct and expressed in tissue culture cells (see §4), comparably low frameshifting efficiencies (namely 0.74 ± 0.13%) were measured. Owing to the low levels involved and the lack of a suitably sensitive antibody to the common N-terminal domain of PA and PA-X, we have not been able to directly measure the frameshifting efficiency in the context of viral infection. Because PA-X is expressed at very low levels during virus infection, we were not able to isolate sufficient quantities from virus-infected cells for mass spectrometric analysis despite multiple attempts. Thus, in order to determine the precise site and direction of frameshifting, we used a construct in which an ORF-encoding green fluorescent protein (GFP) was fused in-frame to the 3′ end of the X ORF (figure 1*b*). Frameshift expression of the construct would result in the transframe fusion PA-X-GFP, which could be affinity-purified on GFP-TRAP beads, while non-frameshift expression would result in a product that does not contain GFP. The construct was expressed in 293T cells, and PA-X-GFP was affinity-purified from cell lysates and resolved by SDS-PAGE. An in-frame control, in which the predicted shift site UCC_UUU_CGU_C was mutated to UCC_UUU_GUC to force expression of PA-X-GFP, was also prepared to show the approximate size at which the frameshift protein should migrate in gels. The wild-type construct produced a specific band migrating at the expected size for PA-X-GFP. A gel slice containing this protein was excised, digested with trypsin, and the resulting peptides were analysed by nano-liquid chromatography tandem mass spectrometry (nano-LC/MS/MS).

Eight separate PA-X-GFP tryptic peptides were identified, including peptides encoded both upstream and downstream of the shift site (figure 1*c*; two of the peptides have overlapping sequence). Importantly, a peptide spanning the shift site itself was identified (figure 1*d*). This peptide, GLWDSFVSPR, defines the shift site (UCC_UUU_CGU) and direction (+1) of frameshifting (figure 1*e*). Molecular ions for GLWDSFVSPR were identified both with and without oxidation at the tryptophan, providing further support for the sequence assignment. No peptide compatible with –2 frameshifting was detected. Formally, the peptide GLWDSFVSPR is compatible with three different models for frameshifting: (i) +1 slippage with UUU in the P-site and an empty A-site; (ii) +1 slippage with UCC in the P-site and an empty A-site; and (iii) tandem +1 slippage with UCC in the P-site and UUU in the A-site. However, consideration of the potential for codon : anticodon re-pairings favours model (i). Both UUU and UUC are translated by a single tRNA isoacceptor whose anticodon, 3′-AAG-5′, has a higher affinity for UUC in the +1 frame than for the zero-frame UUU [32]. By contrast, UCC is expected to be generally decoded by the serine tRNA with anticodon 3′-AGI-5′ (I, inosine), but whether it is decoded by 3′-AGI-5′ or a different serine tRNA when frameshifting occurs, re-pairing to CCU in the +1 frame would involve a mismatch at the first nucleotide position. Moreover, previous experiments showed that mutating UCC to AGC, GGG, CCC or AAA reduced but did not abolish frameshifting, while mutating UUU_CGU to UUC_AGA (with an appropriately positioned 3′ stop codon to prevent non-specific frameshifting elsewhere within the overlap region) knocked out frameshifting [30]. These results are consistent with P-site slippage on UUU_C but argue against P-site slippage on UCC_U, although a low level of slippage on UCC_U cannot be ruled out. Interestingly, a UCC_U tetranucleotide is the site of +1 frameshifting in antizyme expression, although here frameshifting is stimulated, in part, by the presence of a stop codon in the A-site (a role that is unlikely to be substituted by a UUU codon in the A-site) [3].

In other cases of +1 frameshifting, such as in bacteria and yeast, frameshifting is stimulated in part by a slowly decoded A-site codon such as a stop codon or codon whose cognate tRNA is limiting [1,33,34]. At the influenza PA-X shift site, P-site slippage on the UUU_C tetranucleotide may be stimulated by the rare CGU codon in the A-site (CGU is one of the most seldom-used codons in the genomes of mammals and birds—the host species of influenza A virus; table 1 [35]). In support of this, mutating the CGU to the more commonly used arginine codon, CGG, reduced frameshifting by 50 per cent [30]. However, CGU and the more abundantly used codon CGC are expected to be decoded by the same tRNA isoacceptor with anticodon 3′-GCI-5′, and this tRNA species is not obviously limiting in mammals and birds [36,37]. Thus, the role and mode of action of the A-site codon remains uncertain, and conservation of CGU may in part be driven by constraints on the encoded amino acid sequence in the overlapping +1 reading frame.

**Table 1.** Arginine codon usage frequencies (per 1000 codons) in selected organisms.

	*Escherichia coli*	human	chicken	bee	rice	*Arabidopsis*
AGA	2.9	12.2	12.2	22.0	10.5	19.0
AGG	1.8	12.0	11.7	9.1	16.0	11.0
CGU	20.2	4.5	5.4	10.5	7.2	9.0
CGC	20.8	10.4	10.4	5.1	16.1	3.8
CGA	3.8	6.2	5.3	11.4	6.4	6.3
CGG	6.2	11.4	9.7	4.1	13.4	4.9

The role of UCC in the E-site also remains uncertain. In analyses of codon usage in PA, it was observed that the motif UCC_UUU_CGU is extremely highly conserved at the 5′ end of the influenza A virus X ORF, despite the fact that five other codons could potentially be used to encode the serine [30,38]. Moreover, mutating the UCC codon to AGC (serine) or to GGG, CCC or AAA resulted in a 40 to 70 per cent reduction in the frameshifting efficiency [30]. This suggests that UCC plays an important stimulatory role in the E-site. Earlier *in vivo* work on E-site influence (independent of amino acid identity) on stop codon readthrough implies that interactions at that site influence competition for A-site acceptance, but whether this influence acts via the P-site merits investigation [39,40]. Notwithstanding complications due to an interaction with rRNA during bacterial release factor 2 +1 frameshifting, there is evidence in that case for the identity of the E-site codon having an effect on +1 frameshifting. This has been proposed to relate to the speed at which the E-site tRNA is released, with weaker codon : anticodon duplexes being associated with higher levels of frameshifting [41–44]. In an *E. coli* cell-free system, even partially mismatched P-site codon : anticodon interactions, which can be augmented by E-site mismatches, trigger retrospective editing and so influence events in the A-site [45]. A counterpart post-peptide bond effect has not been detected in *S. cerevisiae*, but may exist and involve currently unidentified factors [46,47]. An E-site effect on +1 frameshifting could potentially be influenced by the E-site tRNAs in a proportion of translating ribosomes being near-cognate rather than the standard cognate tRNA. The proposal of an allosteric relationship between release of deacylated tRNA from the E-site being coupled to aminoacyl-tRNA acceptance in the A-site [44] has drawn much criticism [48–51]. On its own, the observed E-site influence on +1 frameshifting could be interpreted as it acting via an effect on the length of the A-site pause that affects the probability of P-site realignment, but a direct effect on P-site codon : anticodon interaction, or rather on the translocating complex, seems more likely.

**Figure 2.**
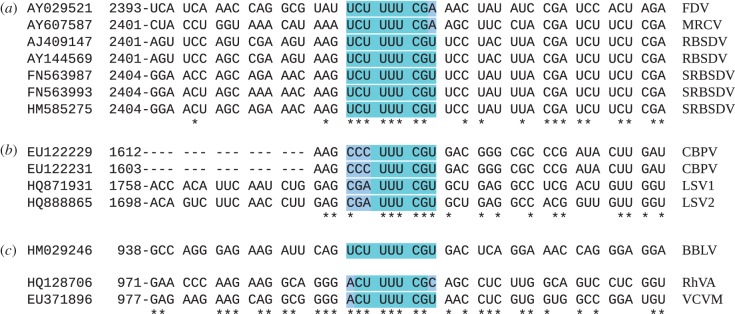
Predicted sites of ‘PAX-like’ +1 frameshifting in (*a*) fijiviruses, (*b*) chronic bee paralysis and Lake Sinai viruses, and (*c*) amalgamaviruses. FDV, Fiji disease virus; MRCV, mal de Rio Cuarto virus; RBSDV, rice black-streaked dwarf virus; SRBSDV, southern rice black-streaked dwarf virus; CBPV, chronic bee paralysis virus; LSV, Lake Sinai virus; BBLV, blueberry latent virus; RhVA, rhododendron virus A; VCVM, *Vicia* cryptic virus M. In all cases, the predicted shift site occurs near the 5′ end of the overlap region between the zero-frame and +1 frame ORFs. Predicted shift sites are highlighted in blue. Dashes in CBPV indicate alignment gaps. Spaces separate zero-frame codons. Note that, downstream of the shift site, the sequences are predicted to be coding in both the zero and +1 frames, and this generally corresponds to enhanced conservation at the nucleotide level. The amalgamavirus sequences are highly divergent, and the precise alignment between BBLV and RhVA+VCVM is ambiguous in this region. GenBank accession numbers, and sequence coordinates of 5′ terminal nucleotides, are indicated at left.

More generally, one might predict a class of +1 frameshift stimulators that comprise a UUU_C P-site slippery sequence and a restricted choice of A- and E-site codons. In eukaryote-infecting viruses, frameshifting by +1 nt has been predicted as the expression mechanism for non-5′-proximal ORFs in the closteroviruses (RdRp), leishmania RNA virus 1 (RdRp), chronic bee paralysis virus and the related Lake Sinai viruses 1 and 2 (RdRp), plant-infecting fijiviruses (Family Reoviridae; P5-2) and members of the proposed family *Amalgamaviridae* of plant viruses (RdRp) (reviewed in [52]). However, in most of these species, the site of frameshifting remained elusive. Characterization of the influenza virus frameshift site now suggests the site of +1 frameshifting in several of these viruses (figure 2). Several of these shift sites are also well supported by comparative genomic analysis [53]. Interestingly, these putative shift sites all seem to show a preference for A-site CGN codons, as opposed to other CNN codons. As in PA-X expression, it is likely that the efficiency of frameshifting at such sites is low. However, these levels may be completely compatible with the expression level requirements of some viruses (cf. –1 frameshifting for polymerase expression in *S. cerevisiae* totivirus L-A, where the ratio of Gag-Pol to Gag in the virion is of order 1–2% and, correspondingly, the frameshifting efficiency is around 1.8%) [54]. Whether similar motifs are functionally used for cellular gene expression remains to be seen.

## 4. Methods

### 4.1. Dual luciferase reporter constructs and assays

Sequences encompassing the frameshift site (97 nt 5′+UCC_UUU_CGU+100 nt 3′) were generated using overlapping synthetic oligonucleotides and cloned into pDluc, a derivative of the dual luciferase reporter p2luc vector [55,56]. The 3′ firefly luciferase ORF is in the +1 frame relative to the 5′ renilla luciferase ORF, so that frameshifting within the inserted sequence results in a fusion of both ORFs. An in-frame control, which was identical except that the UCC_UUU_CGU_C shift site was mutated to UCC_UUC_GUC, was also constructed. All constructs were verified by DNA sequencing. Frameshift assays were performed as described previously [55,57]. Frameshift efficiencies were calculated as (firefly activity/renilla activity) for the frameshift sequence normalized by (firefly activity/renilla activity) for the in-frame control sequence. Means and standard errors were calculated based on four to six independent transfections. Owing to the low frameshifting efficiencies involved, a low level of background firefly activity (e.g. owing to cryptic splice sites, cryptic promoters, degraded transcripts or non-specific IRES activity) was a potential issue. To control for this, firefly and renilla activities were also measured for a corresponding shift-site mutant sequence (UUU_CGU mutated to UUC_AGA), and the ratio was subtracted from the ratio measured for the WT sequence. (It should be noted that independent initiation of the downstream reporter is not an issue for the previous frameshift efficiency measurements in rabbit reticulocyte lysates, where radiolabelled translation products could be visualized via SDS-PAGE.)

### 4.2. Protein purification

To create the PA-X-GFP expression construct, the nucleotide sequence corresponding to the coding region of PA-X, minus the X-ORF stop codon, was amplified from a A/Brevig Mission/1/1918 (H1N1) segment 3 reverse genetics plasmid [58] and cloned into pEGFP-N1 using standard techniques (forward primer 5′-GCCACCGGTACCATGGAAGACTTTGTGCGACAATG-3′; reverse primer 5′-GCCACCACCGGTCTTCTTTGGACATTTGAGAAAGC-3′). To avoid PA-X auto-repressing its own synthesis, the PA endonuclease active site was inactivated via the mutation D108A [30]. The GFP-initiating ATG was also mutagenized (ATG to TG) to bring the downstream GFP ORF in-frame with the +1-frameshifted X-ORF and to prevent downstream GFP initiation (forward primer 5′-CCGGTCGCCACCTGGTGAGCAAGG-3′; reverse primer 5′-CCTTGCTCACCAGGTGGCGACCGG-3′). For the in-frame control construct, site-directed mutagenesis was used to delete the cytosine that is skipped during frameshifting, using standard techniques. Constructs were transfected into 293T cells using Lipofectamine 2000 (Invitrogen), according to the manufacturer's instructions. After incubation for 48 h, cells were lysed and GFP-TRAP-A purification (Chromotek) was performed, as previously described [59]. The GFP-TRAP bound fraction was resolved by SDS-PAGE, and polypeptides were visualized by silver staining.

### 4.3. Mass spectrometric analysis

Gel slices containing proteins of interest were excised, digested with trypsin, and analysed by nano-LC/MS/MS. All mass spectra were acquired with an LTQ-FT instrument (ThermoElectron). nano-LC with nano-electrospray was used with a 75 μm ID column (C18) and an acetonitrile gradient (0.1% formic acid). Primary mass spectra of peptide molecular ions, primarily observed at +2 charge states, were obtained in the FT-ICR (Fourier transform ion cyclotron resonance) part of the instrument. All peptide masses assigned were better than 2 ppm mass error compared with theoretical values. Both oxidized (i.e. addition of O, occurring at methionine, tryptophan or histidine) and non-oxidized forms were identified for many peptides. Oxidation of peptides is a common occurrence observed during ionization with electrospray, but oxidation can also be present as a post-translational event. Peptide sequence information was acquired using MS/MS with the ion-trap part of the LTQ-FT instrument using collision-induced dissociation fragmentation of selected peptide masses. Peptides were assigned based on combined evidence of the molecular ions and MS/MS sequence. Searches of custom sequence databases were performed with Mascot [60], using strict parameters to generate high-confidence assignments, and, in addition, all primary and MS/MS data were reviewed manually for accuracy.

## 5. Acknowledgements

A.E.F. is supported by the Wellcome Trust (088789). J.F.A. is supported by Science Foundation Ireland (08/IN.1/B1889). P.D. is supported by Institute Strategic Grant Funding from the UK Biotechnology and Biological Sciences Research Council and the U.K. Medical Research Council (G0700815). B.W.J., P.D. and J.K.T. are also thankful for the support of the NIH-Oxford-Cambridge Research Scholars programme.
